# The chemo-prophylactic efficacy of an ethanol *Moringa oleifera* leaf extract against hepatocellular carcinoma in rats

**DOI:** 10.1080/13880209.2017.1306713

**Published:** 2017-03-27

**Authors:** Kadry M. Sadek, Tarek K. Abouzed, Reham Abouelkhair, Sherif Nasr

**Affiliations:** a Department of Biochemistry, Faculty of Veterinary Medicine, Damanhur University, Egypt;; b Department of Biochemistry, Faculty of Veterinary Medicine, Kafr elshiekh University, Egypt;; c Department of Nutrition, Faculty of Veterinary Medicine, University of El-Sadat City, Egypt;; d Department of Molecular Biology and Genetic Engineering, Faculty of Veterinary Medicine, Damanhur University, Egypt

**Keywords:** Apoptosis, gene expression, oxidative DNA

## Abstract

**Context:** Hepatocellular carcinoma (HCC) is among the most well-known threatening tumours around the world, and the outlook remains bleak. *Moringa oleifera* Lam. (Moringaceae) exhibits antitumor, antioxidant and hepatoprotective properties.

**Objectives:** To assess the chemo-prophylactic proficiency and other likely activities of *Moringa oleifera* leaf ethanol extract (MOLEE) against diethyl nitrosamine (DEN)-induced HCC.

**Materials and methods:** Wistar rats were gastrogavaged with MOLEE (500 mg/kg) for one week and then gastrogavaged with MOLEE and DEN (10 mg/kg) for the following 16 weeks. The progressions of the histological components, serum biomarkers and oxidation of DNA of the liver tissues were resolved to assess the prophylactic impacts. The lipid oxidative biomarker, the cancer prevention agent status and apoptotic proteins were surveyed to assess the potential mechanisms.

**Results:** The MOLEE LD50 was estimated to be 5585 mg/kg. MOLEE (500 mg/kg) administration fundamentally repressed the expansion event of knobs and the normal knob number per knob-bearing livers prompted by DEN, enhanced hepatocellular appearance and altogether significantly decreased (*p* < 0.05) DEN-induced elevations in serum biochemical records and hepatic 8-hydroxy-2-deoxyguanosine (8-OHdG) levels by 29%. The robotic studies found that MOLEE disrupted the DEN-activated oxidative reactivity damage in rats by 46.8%. Curiously, the expression of Bcl-2, Bcl-xl and β-arrestin-2 were fundamentally diminished (*p* < 0.05); however, the expression of Bax and caspase-3 were essentially (*p* < 0.05) upregulated.

**Discussion and conclusions:** The outcomes presume that MOLEE inspired critical defensive impacts against DEN-induced hepatocarcinogenesis that might be identified with the implementation of antioxidant activity and actuation of apoptosis.

## Introduction

Liver cancer is the most prominent among the successive reasons for death throughout the world (Jemal et al. 2011). Hepatocellular carcinoma (HCC) is the widely recognized danger to the liver, and the third most common reason for tumour death around the world, and there are few successful remedial choices for this serious malady (Ferenci et al. 2010). The dominant part of HCCs occur in cirrhotic livers following a long duration of constant liver aggravation brought on by viral hepatitis and alcoholic and nonalcoholic steatohepatitis (Asahina et al. 2010). HCCs diagnosed after the onset of side effects have poor prognoses (0–10% 5-year survival) (Llovet et al. 2003). Environmental cancer-causing agents are a main consideration that can prompt the emergence of liver malignancy. Diethyl nitrosamine (DEN) is generally widespread in nature and is present in various items, for example, cheddar, handled meats, mixed drinks, tobacco items and restorative items. DEN was observed to be one of the natural cancer-causing agents (Amin et al. 2011). DEN can prompt hepatic cancer in all types of animals, including humans (Loeppky 2009).

The customary treatments for liver malignancy comprise radiotherapy, chemotherapy, surgical mediation and removal, and are not prescribed without the proper workup and diagnosis because of the severe side effects and the poor outlook regarding HCC. If these treatments fail, then liver transplantation, as the last resort, will hopefully become the effective treatment for hepatic cancer. Be that as it may, the constrained accessibility of organs precludes this option for many people with HCC, and the high risk of tumour recurrence after transplantation further complicates the efficacy of this treatment strategy (Tabone & Pellicano 2006). Thus, the discovery of potential therapeutic targets that cause less toxic side effects are critical for inhibiting or delaying hepatic cancer.

*Moringa oleifera* Lam. (Moringaceae) is native to India, Africa, Arabia, Southeast Asia, South America, and the Pacific and Caribbean Islands (Iqbal & Bhanger 2006). The leaves of *Moringa oleifera* can be eaten crisp or cooked, and reports have shown that the leaves can be put away as a dried powder for a long time with no considerable loss of wholesome quality (Arabshahi et al. 2007). *Moringa oleifera* leaves have been found to have the same powerful antioxidant agents such as vitamins C, E and A in oranges, pomegranates and carrots, as well as caffeoylquinic acids, carotenoids (i.e., lutein and α- and β-carotene), kaempferol, quercetin and rutin (Smolin & Grosvenor 2007). *Moringa oleifera* oil and its micronutrients exhibit antitumor, antioxidant, antiepileptic, antidiuretic, anti-inflammatory, hepatoprotective and antidiabetic properties (Sreelatha & Padma 2010). Accumulating evidence found that overproduction of reactive oxygen species (ROS) assumes a key role in the aetiology of HCC. ROS can bring about oxidative harm to DNA that encourages the advancement of HCC (Lin et al. 2007). Additionally, dysregulated apoptosis is identified with hepatocarcinogenesis that is induced by numerous chemicals, including DEN (Finnberg et al. 2000). Since *Moringa oleifera* leaf ethanol extract (MOLEE) has efficacious antioxidant capacity and the constituents of MOLEE have proapoptotic effects in numerous growths including cancer (Smolin & Grosvenor, 2007; Sreelatha & Padma 2010), MOLEE was investigated to determine if it could hinder DEN-prompted hepatocarcinogenesis in a rodent model.

## Materials and methods

### Materials

Crisp leaves of the drumstick tree (*Moringa oleifera*) were obtained from a neighbourhood garden at the Badr Center, Behera governorate, Egypt. DEN was obtained from Sigma Chemical Co. (St. Louis, MO). The DNeasy tissue kit was obtained from the Promega Corporation (Madison, WI). The 8-hydroxy-2-deoxyguanosine (8-OHdG) ELISA kit was provided by Cell Biolabs, Inc. (San Diego, CA). Commercial antioxidant assay kits were purchased from Nanjing Jiancheng Bioengineering Institute (Nanjing, China). All other reagents were of analytical, high-performance liquid chromatography (HPLC) grade or the best available pharmaceutical grade.

### Preparation of the extract

The plant material (1 kg) was air-dried at room temperature. The dried leaves were ground into a fine powder and stored in a water/air proof holder. The resultant dry powder (300 g) was soaked in 500 mL of ethanol (95%) for 24 h in a percolator. After 24 h, moderate permeation was permitted, and the concentrate was gathered in Petri dishes. The concentrate was amassed in a vacuum utilizing a revolving streak evaporator. The net yield of concentrate was 30.5 g (10.16% w/w). The rough concentrate was suspended in refined water preceding administration to the rats.

### Extract authentication

MOLEE was verified by Reham Abouelkair, Department of Nutrition, Faculty of Veterinary Medicine, University of El-Sadat City, Egypt (Tables 1–3).

**Table 1.** Chemical composition of *Moringa oleifera* leaves ethanol extract.

Chemical composition	MOLEE
Moisture Conc. (g/100 g)	10.74 ± 0.05
Fiber Conc. (g/100 g)	11.23 ± 0.16
Fat Conc. (g/100 g)	7.76 ± 0.21
Protein Conc. (g/100 g)	9.38 ± 0.23
Sugar Conc. (g/100 g)	56.33 ± 0.27
Ash Conc. (g/100 g)	4.56 ± 0.13
Energy (kCal)	332.68 ± 0.06
Mg Conc. (mg/100 g)	25.64 ± 0.25
Zn Conc. (mg/100 g)	1.63 ± 0.021
Mn Conc. (mg/100 g)	5.21 ± 0.12
Cu Conc. (mg/100 g)	0.88 ± 0.52
Vitamin C Conc. (mg/100 g)	245.13 ± 0.46
Vitamin A (β- Carotene) Conc. (mg/100 g)	13.48 ± 0.51
Vitamin E Conc. (mg/100 g)	16.80 ± 0.24
Total phenolic content (mg GAE/g)	48.35 ± 0.05
Total flavonoids (mg/g)	35.64 ± 0.07

**Table 2.** Bio-active compounds of *Moringa oleifera* ethanol extract.

RT	Compound name	Area %	Molecular formula	Molecular weight
16.35	Caryophyllene	12.48	C_15_H_24_	204
16.61	Eugenol	45.15	C_10_H_12_O_2_	164
16.61	Phenol, 2-methoxy-4-(2-propenyl)-	45.15	C_10_H_12_O_2_	164
22.30	Phenol, 2-methoxy-4-(2-propenyl)-acetate	5.06	C_12_H_14_O_3_	206
29.24	Pentadecanoic acid	1.27	C_17_H_34_O_3_	270
30.09	Hexadecanoic acid, 2,3-dihydroxy propyl ester, 14-methyl-, methyl ester	6.87	C_19_H_38_O_4_	330
32.11	*trans*-13-Octadecanoic acid, methyl ester	4.40	C_19_H_36_O_2_	296
33.34	13-Heptadecyn-1-ol	2.45	C_17_H_32_O	252
33.73	9,12,15-Octadecatrienoic acid, 2,3- dihydroxy propyl ester	2.35	C_21_H_36_O_4_	352

**Table 3.** The bioactive oil of MOLEE.

Component	Ri^a^	Ri^b^	Identification^c^	%
Oxygenated monoterpenes				
Linalool	1033	1450	1,2,3	t
α-Terpineol	1123	1608	1,2,3	t
Phenolic compounds				
*p*-Vinylguaiacol	1212	1836	1,2	t
Oxygenated sesquiterpene				0.8
*cis*-Dihydroagarofuran	1416		1,2	0.2
Eudesm-11-en-4-α,6α-diol	1707		1,2	0.6
Hydrocarbons				90
1-Octadecene	1145		1,2	0.3
*n*-Hexadecanol	1153		1,2,3	0.7
Nonadecane	1161		1,2	0.6
1-Eicosene	1172		1,2	0.4
*n*-Octadecanol	1188		1,2,3	0.5
Heneicosane	1191		1,2	1.5
Cyclopentadecanol	2121		1,2,3	1.3
1-Docosene	2440		1,2	0.7
*cis*-9-Eicosen-1-ol	2566		1,2,3	10.5
Tricosane	2675		1,2,3	15.0
Tetracosane	2689		1,2,3	20.1
Pentacosane	2722		1,2,3	15.5
Hexacosane	2798		1,2,3	10.5
Heptacosane	2826		1,2,3	12.0
Octacosane	2843		1,2,3	1.4
Nonacosane				
Triacontane				
Others				0.5
Hexenyl propanoate	1167		1,2	0.5
Phenylethyl alcohol	1176		1,2	t
Pseudo Phytol	1188		1,2	t

a Kovats retention index on HP-5 MS column.

b Kovats retention index on HP Innowax.

c 1 = Kovats retention index, 2 = mass spectrum, 3 = co-injection with authentic compound; t = trace, less than 0.1%.

### Animals and treatments

Forty male Wistar male rats weighing 140–160 g were given by the Faculty of Science, Tanta University. The rats received humane care as per the rules of the National Institutes of Health (NIH) for ethical treatment and management of laboratory animals (NIH 1996). The rats were housed separately, and a standard diet and water were supplemented *ad libitum*. A constant temperature of 25 ± 3 °C and 50% relative humidity on a 12 h light/dark cycle was maintained at the animal room. Following two weeks on the basal eating routine, the animals were arbitrarily separated into four groups (*n* = 10). The rats in the MOLEE groups were pretreated with MOLEE (500 mg/kg bw) by gavage for 7 days, and the rats in the control and DEN groups received equivalent volumes of corn oil. At that point, all animals aside from those in the control group received oral DEN (10 mg/kg bw) for 16 consecutive weeks, and the rats in MOLEE groups were persistently directed MOLEE until the end of the investigation. The dose of MOLEE depended on the results of the previous study of Sadek (2014), and the dosage of DEN was chosen based on the past reports of Cui-Li et al. (2012). Body weights were measured every week, and doses were changed as needed. Following 16 weeks of DEN organization, all rats were anesthetized 24 h after the last treatment. Blood was gathered (3 mL) after cervical beheading and centrifuged at 704*g* for 15 min at 4 °C to obtain the serum. Liver tissue was extracted and weighed. The quantities of knobs on the liver surface were checked. A small part of the liver was settled in paraformaldehyde (10%) for histopathological examination. The other part of the liver tissue was immediately frozen in liquid nitrogen before storage at −80 °C.

### Liver morphology and histology

The quantities of knobs in the livers were checked in 3 mm cross-areas, and the knob frequencies and normal knob numbers per knob-bearing liver were computed for all groups. For the histological examination, ∼5 μm paraffin-embedded areas were readied, deparaffinized, rehydrated, stained with hematoxylin and eosin (H&E), and visualized with an Olympus AX70 magnifying lens (Tokyo, Japan).

### Serum biochemical assays

The activities of serum enzymes, albumin (ALB), globulin (GLB) and total protein (TP) were resolved utilizing Unico 2100 UV-Spectrophotometers, ELx800 Absorbance Microplate Reader and performed by directions of its pack.

### Quantification of 8-OHdG levels in liver DNA with competitive ELISA

The DNA was separated from solidified liver tissues utilizing a DNeasy tissue unit. The 8-OHdG levels in the liver DNA tests were examined utilizing an ELISA unit. Briefly, the 8-OHdG immune response and the specimen were added to an ELISA plate that had been precoated with 8-OHdG. The 8-OHdG in the specimen competed with the 8-OHdG bound to the plate for the 8-OHdG counter acting agent restricting destinations. The normal groupings of 8-OHdG per microgram of DNA for every group were computed for every example.

### Enzyme-linked immunosorbent assay (ELISA) for AFP and CEA

Assessment of the levels of the tumour markers α-fetoprotein (AFP) and carcinoembryonic antigen (CEA) were carried out on solid-phase enzyme-linked immunosorbent assays (ELISAs) that were performed using UBIMAGIWELL (USA) enzyme immunoassay kits.

### Liver antioxidant statuses

The liver tissue was homogenized in 9 volumes of ice-cold buffer (pH 7.4) containing 0.01 M Tris-HCl, 0.1 mM EDTA-2Na, 0.01 M saccharose and 0.8% saline. The homogenates were centrifuged at 1000*g* for 20 min at 4 °C. The supernatants were collected and stored at −80 °C for the antioxidant assays using commercial assay kits according to the manufacturer’s instructions.

### RNA extraction and cDNA synthesis

Total RNA was separated from rodent livers utilizing Trizol reagent (Invitrogen Corp., Carlsbad, CA) as per the manufacturer’s guidelines. The RNA pellet was broken up in DEPC water. The concentration and purity of the aggregate RNA were measured utilizing a Nanodrop spectrophotometer (Nanodrop 2000c, Thermo Scientific, Waltham, MA) and Agilent 2100 Bioanalyzer (Agilent Technologies, Boblingen, Germany). Correlative DNA was blended utilizing the RevertAid TM First Strand cDNA Synthesis Kit as per the manufacturer’s convention.

### Real-time PCR analysis

The gene expression of hepatic apoptotic-related proteins was determined with real-time PCR. The primers were synthesized by Sangon Biotech Co., Ltd. (Shanghai, China) (Table 4). All PCR reactions were performed utilizing Maxima SYBR Green qPCR Master Mix and were completed under the accompanying conditions utilizing a MasterCyclerTM Eppendorf realplex 4 (Eppendorf, Westbury, NY): initial denaturation at 95 °C for 10 min, followed by 40 cycles of 20 s at 94 °C, 35 s at 62 °C and 35 s at 72 °C. Every specimen was analyzed in triplicate. The distinctions in quality expression between the groups were determined utilizing the △△Ct (process duration, Ct) strategy (Livak & Schmittgen 2001) and were standardized to glyceraldehyde-3-phosphate dehydrogenase (GAPDH) and are presented as relative mRNA levels compared to the controls.

**Table 4.** Nucleotide sequences of the primers used in RT-PCR

Gene symbol	GenBank accession no.	Primer (5′→3′)	
Bcl-2	NM_016993.1	F:	CCCCAGAAGAAACTGAACC
		R:	GCATCTCCTTGTCTACGC
Bcl-xL	XM_017591479.1	F:	CGTGGAAAGCGTAGACAAGG
		R:	CAACAACCATGCCAGGAGAC
β-arrestin-2	NM_012911.1	F:	CCACGTCACCAACAATTCTG
		R:	TTGGTGTCTTCGTGCTTGAG
Bax	NM_017059.2	F:	GTTGCCCTCTTCTACTTTGC
		R:	ATGGTCACTGTCTGCCATG
caspase-3	NM_012922.2	F:	CTGGACTGCGGTATTGAGAC
		R:	CCGGGTGCGGTAGAGTAAGC
GAPDH*	NM_017008.4	F:	TCAAGAAGGTGGTGAAGCAG
		R:	AGGTGGAAGAATGGGAGTTG

*Housekeeping gene

### Statistical analysis

SPSS 13.0 (Chicago, IL) was used for the statistical analysis. A chi-square test was used for the analysis of nodule incidence. The other data are expressed as the mean ± the SEs and were analyzed with one-way ANOVAs followed by Tukey’s tests for the multiple comparisons. Differences were considered significant at the *p* < 0.05 level.

## Results

### Changes in body and relative liver weights

**Figure 1.**
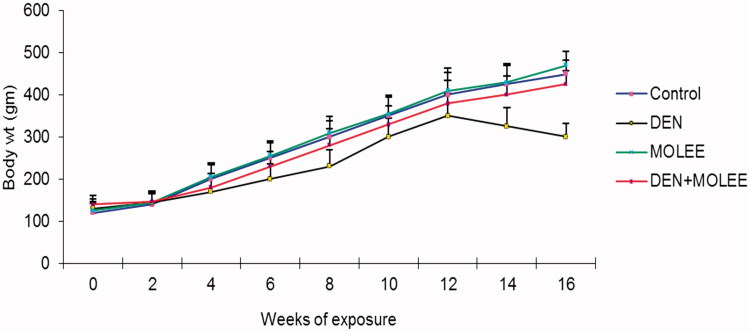
Changes of rat body weight. The data of body weight are presented as the mean ± S.E. (*p* < 0.05).

Figure 1 delineates the impact of MOLEE on body weight changes induced by DEN. In the principal week of post DEN treatment, the rats started to demonstrate moderate development. A short time later, with ceaseless DEN introduction, the mean weight pick-up diminished slowly. Following 14 weeks of presentation, the body weight in the DEN group started to show negative development. Toward the end of 16 weeks, the body weight of the rats in the control group expanded to ∼3-overlay their underlying qualities, though the weight of the rats in the DEN group were ∼2.13-fold their underlying qualities. Again, MOLEE treatment fundamentally re-established the addition of the body weight of the rats compared to the DEN group (Figure 1). Table 5 shows the last body and liver weights of the distinctive groups of rats. The relative liver weight in the DEN group was expanded compared to that in the control group (*p* < 0.05). The administration of 500 mg/kg MOLEE altogether diminished the relative liver weight compared to the DEN group (*p* < 0.05).

**Table 5.** Effect of MOLEE and DEN on body and liver weights of different groups of rats.

Groups	Final body weight (g)	Liver weight (g)	Relative liver weight
Control	450 ± 37^a^	16 ± 2.32^b^	0.0355^c^
DEN	320 ± 41^c^	29 ± 2.54^a^	0.0906^a^
MOLEE	465 ± 29^a^	14 ± 2.54^b^	0.0297^c^
DEN + MOLEE	425 ± 23^ab^	18 ± 1.65^b^	0.0423^b^

Means within the same column carrying different letters are significantly different (*p* < 0.05).

### Appearance and histological changes in the liver

**Figure 2.**
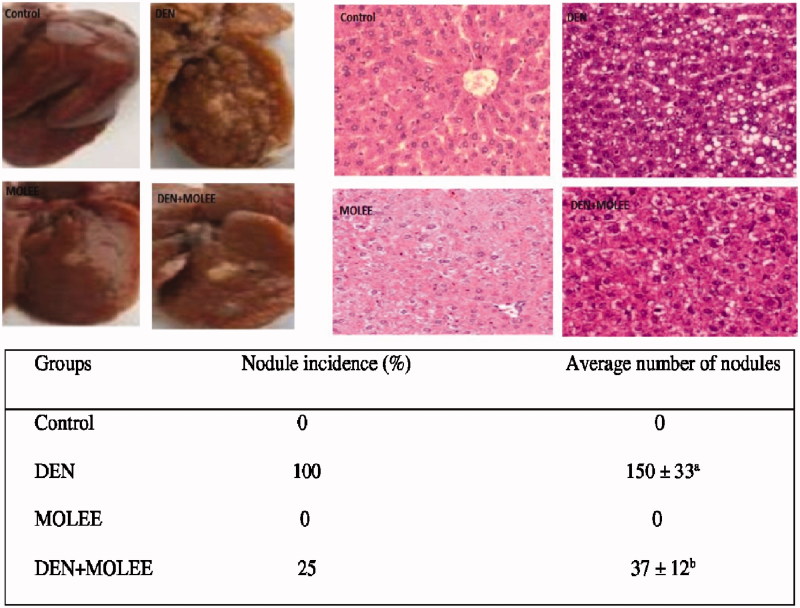
Effect of MOLEE on hepatic morphology, histology (HE staining, ×200) and changes of nodule incidence and average number of nodules per nodule-bearing liver in rats. The data are presented as the mean ± S.E. (*p* < 0.05).

The morphology of the livers in the control group was typical, and there were no noticeable, visible knobs. The rats in the DEN group indicated amplified livers. The event of knobs in the DEN group was 100%, and the most extreme breadth of the knobs was ∼10 mm. Moreover, clearly necrotic ranges were available in the livers of the DEN-treated rats. Shockingly, huge diminishments in liver broadening, knob events and normal knob numbers per knob-bearing liver were recognized in the MOLEE-treated rats compared with the DEN-group rats (Figure 2). The histological photos of each group’s livers are available in Figure 2. The livers of the control group indicated typical hepatic architecture. The hepatocellular architecture in the DEN group was totally destroyed and a considerable measure of hyperplastic knobs was found. The nuclei were noticeable and involved most of the cell space. These histopathological appearances were extraordinarily enhanced in the livers of the rats that were treated with MOLEE. In other words, histological element in the MOLEE group (500 mg/kg) showed a huge change in liver architecture compared to the DEN group.

### Serum biomarkers assays

The activities of serum ALT, AST, ALP, LDH and GGT were increased in the DEN group compared to the control group (*p* < 0.05), which affirmed liver harm and preneoplastic lesions. Despite what might be expected, the serum albumin level was diminished, and the globulin level was unaltered in the DEN group compared with the control group. The A/G proportion was lessened by DEN treatment. These unfavourable effects of DEN were relieved and deflected by MOLEE (Tables 6 and 7).

**Table 6.** Effect of MOLEE and DEN on serum specific liver enzyme activities in rats.

Groups/parameters	ALT (IU/L)	AST (IU/L)	GGT (IU/L)	LDH (IU/L)	ALP (IU/L)
Control	34.5 ± 6.2^c^	41.4 ± 5.2^c^	59.8 ± 6.93^c^	457.5 ± 23.7^c^	127.6 ± 14.6^b^
DEN	135.8 ± 21.8^a^	148.2 ± 11.5^a^	99.3 ± 8.81^a^	885.41 ± 29.8^a^	194.2 ± 17.9^a^
MOLEE	32.3 ± 5.1^c^	29.1 ± 5.8^d^	55.5 ± 7.81^c^	348.2 ± 31.9^d^	103.2 ± 19.3^c^
DEN + MOLEE	90.3 ± 10.6^b^	100.5 ± 13.8^b^	73.4 ± 6.81^b^	561.2 ± 66.8^b^	133.4 ± 11.8^b^

Means within the same column carrying different letters are significantly different (*p* < 0.05).

**Table 7.** Effect of MOLEE and DEN on protein pattern and MDA in rats.

Groups/parameters	Total protein (g/dL)	Albumin (g/dL)	Globulin (g/dL)	A/G ratio	MDA (nmol/g)
Control	8.3 ± 0.7^a^	5.2 ± 0.2^a^	3.1 ± 0.02	1.76 ± 0.002	66.34 ± 6.78^c^
DEN	4.7 ± 0.08^c^	1.8 ± 0.03^c^	2.9 ± 0.03	0.62 ± 0.001	351.51 ± 24.67^a^
MOLEE	8.5 ± 0.3^a^	5.4 ± 0.4^a^	3.1 ± 0.04	1.74 ± 0.002	38.21 ± 7.83^d^
DEN + MOLEE	6.4 ± 0.09^b^	3.1 ± 0.1^b^	3.3 ± 0.01	0.93 ± 0.003	187.71 ± 18.58^b^

Means within the same column carrying different letters are significantly different (*p* < 0.05).

### Levels of 8-OHdG in liver DNA

**Figure 3.**
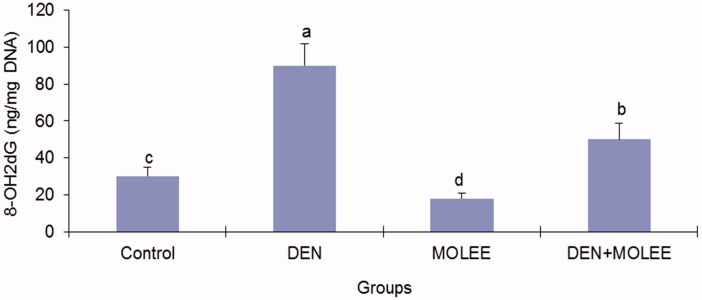
The content of 8-OHdG in liver DNA. The data are presented as the mean ± S.E. Columns with different letters are significantly different (*p* < 0.05).

As shown in Figure 3, following 16 weeks of DEN treatment, the 8-OHdG level was increased by 3-fold (*p* < 0.05) compared to the level observed in the control group. While this augmentation in 8-OHdG induced by DEN was fundamentally diminished in the MOLEE-treated groups, this decrease achieved 29% (*p* < 0.05) in the 500 mg/kg group.

### Effects of MOLEE on tumour markers

**Figure 4.**
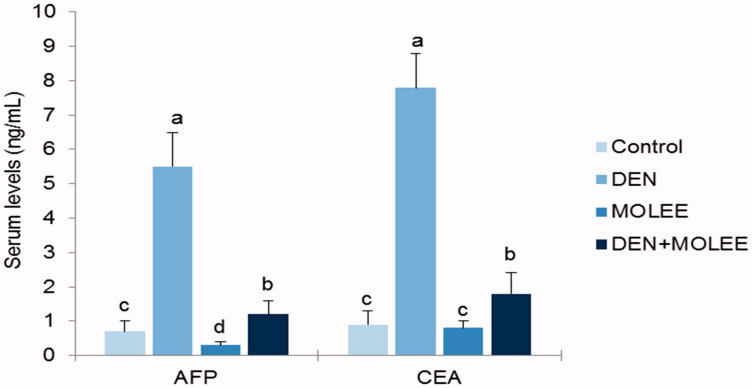
Effect of MOLEE on the level of AFP and CEA in the serum of control and experimental rats. The data were presented as the mean ± S.E. Columns with different letters are significantly different (*p* < 0.05). AFP and CEA levels are expressed as ng/mL.

The impacts of MOLEE on tumour markers (i.e., AFP and CEA) in the sera of the control and experimental animals are delineated in Figure 4. Tumour marker levels altogether expanded (*p* < 0.05) in group 2 (DEN) animals compared to control group animals. Interestingly, the administration of MOLEE essentially lessened tumour marker levels in group 4 (DEN + MOLEE) test animals compared to aggregate 2 animals.

### Changes in lipid peroxidation (LPO) and antioxidative status

With respect to parts of ROS in the development of DEN-prompted HCC, it was imperative to assess the adjustments in the hepatic antioxidant system, which is displayed in Tables 7 and 8. DEN significantly increased the malondialdehyde (MDA) level, and this increase was notably stifled by MOLEE treatment. Compared with the DEN group, the MDA level was diminished by 46.8% (*p* < 0.05) in the 500 mg/kg MOLEE group. Moreover, DEN brought on critical declines in GSH levels and weakened the activities of SOD, GST, CAT and GPx. Compared with the DEN group, the 500 mg/kg MOLEE group displayed noteworthy increments of 183, 190, 167, 139 and 154% in hepatic GSH levels and the exercises of SOD, GST, CAT and GPx, respectively.

**Table 8.** Effect of MOLEE and DEN on oxidant/antioxidant status in rats.

Groups/parameters	GSH (mg/g)	GPx (IU/g)	CAT (U/mg protein)	SOD (U/mg protein)	GST (U/mg protein)
Control	104.69 ± 13.87^b^	52.56 ± 4.27^a^	87.33 ± 6.68^a^	23.56 ± 4.51^b^	31.39 ± 3.91^b^
DEN	43.54 ± 5.76^d^	31.57 ± 4.43^b^	41.47 ± 7.18^c^	11.36 ± 2.37^c^	13.71 ± 3.42^d^
MOLEE	121.35 ± 17.62^a^	53.92 ± 7.92^a^	85.88 ± 9.09^a^	39.95 ± 9.99^a^	53.47 ± 8.32^a^
DEN + MOLEE	79.87 ± 7.87^c^	48.88 ± 5.73^a^	57.89 ± 5.27^b^	21.67 ± 7.80^b^	23.59 ± 4.87^c^

Means within the same column carrying different letters are significantly different (*p* < 0.05).

### MOLEE enhanced apoptosis in the livers of DEN-induced rats

**Figure 5.**
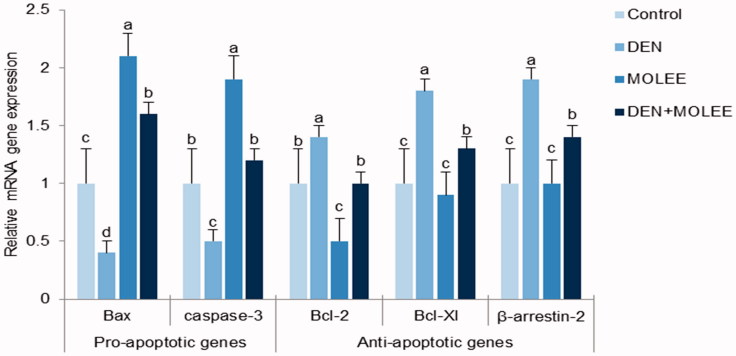
Effect of MOLEE and DEN on the mRNA levels of Bcl-2, Bcl-XL, Bax, caspase-3 and β-arrestin. The mRNA levels were quantified with GAPDH as an internal control. The data were presented as the mean ± S.E. Columns with different letters are significantly different (*P* < 0.05).

Because of the genuine data that says disturbance of apoptosis is a pivotal reason for the organization of HCC (Fabregat 2009), we needed to assess the mRNA expression levels of apoptosis-related proteins. As represented in Figure 5, quantitative real-time PCR results indicated critical increases in the mRNA expression levels of the anti-apoptotic proteins Bcl-2, Bcl-xl and β-arrestin-2 in the DEN group compared with the control group (*p* < 0.05). Furthermore, noteworthy reductions in the mRNA expression levels of Bax and caspase-3 were seen in the DEN group. Thus, the Bcl-2/Bax proportion remained altogether higher in the DEN group than in the control group. Then again, the administration of MOLEE clearly diminished the expressions of Bcl-2, Bcl-xl and β-arrestin-2 and expanded the expressions of Bax and caspase-3. Shockingly, the Bcl-2/Bax proportions were fundamentally diminished in the MOLEE groups compared with the DEN group.

## Discussion

HCC is a noteworthy issue in developed nations as well as in the larger part of undeveloped nations. HCC are initiated by poisonous modern chemicals, air contaminations, nourishment added substances and fungal poisons (Arya et al. 1988).

In the present study, MOLEE was found to fundamentally diminish the numbers and sizes of knobs, enhance the histopathological features and serum biomarkers and keep the DNA oxidation induced by DEN. We likewise observed that MOLEE mitigated the hepatic antioxidant status and regulated the process of apoptosis in the liver tissue. These outcomes give solid data that MOLEE altogether restrained the movement of DEN-activated hepatic tumours. The nodular number was found to be proportional with HCC in test models (Bishayee & Chatterjee 1995); subsequently, the counteractive action of knob improvement and events by MOLEE as shown in the present investigation is required for HCC inhibition.

Biochemical marker enzymes are utilized for screening, especially screening of malignancy conditions, to empower differential judgments, hypotheses, progress observing, and appraisal of reactions to treatment (Mc-Intrye & Rosalki 1992). These catalysts are more exceptional, and changes in their activities mirror the impacts of the multiplication of cells with development potential and their metabolic turnover. Increments in the activities of these enzymes have been exhibited to associate well with the quantities of changed cells in malignancy conditions (Kamdem et al. 1982). The serum activities of transaminases and ALP are utilized as indices of liver damage (Shaarawy et al. 2009). LDH and GGT are a commonplace and sensitive marker of strong neoplasms (Lippert et al. 1981). The information of the present study uncovered significant inhibitory impacts of MOLEE on DEN-prompted enzymatic activity builds, which affirmed that MOLEE smothered DEN-induced liver harm.

The damage of DNA induced by oxidation might be a vital deciding variable that prompts tumours (Hagen et al. 1994). DEN is known as intense hepatocarcinogen in rats that may inspire its activity through changing DNA structure, particularly by means of the development of alkyl DNA adducts and enlistment of chromosomal abnormalities, in addition to micronuclei in the liver tissue (Al-Rejaie et al. 2009). Saalu et al. (2011) reported that *Moringa oleifera* contains fundamental antioxidant and phenolic compounds that ensure intensification against oxidative changes evoked by harmful materials and certain antineoplastic specialists. To facilitate the assessment of the parts of MOLEE in the inhibition of DEN-induced advancement of HCC, we inspected the levels of 8-OHdG, which is a DNA base-changed item created by responsive oxygen species and a decent marker of oxidative DNA harm because of maturing and growth and other degenerative diseases (Tsubota et al. 2008). The information clearly demonstrated that MOLEE administration fundamentally diminished the levels of 8-OHdG in liver DNA compared with the levels observed in the DEN group, which proposes that MOLEE altogether forestalled DEN-induced DNA damage.

α-Fetoprotein (AFP) is an oncofetal protein in the serum that is gradually evaporated amid advancement until totally dissolved in healthy grown-ups (Sell et al. 1983). The introduction of rats to specific cancer-causing agents, for example, DEN, has been found to bring about rise in coursing AFP levels (Becker & Sell 1979). Carcinoembryonic antigen (CEA) is a member of the immunoglobulin supergene family and a 180–200 kDa highly glycosylated protein that is utilized clinically as a tumour marker for diagnosing the recurrence of numerous sorts of cancers (Zimmer & Thomas 2001). In the present study, increments in serum AFP and CEA levels upon DEN induction were associated with increases in tumour development. Decrease in such tumour markers after MOLEE administration may have been because of abatements in the rate of tumour generation.

The liver is the significant site of the DEN digestion system, and the creation of ROS in the liver is recognized as a critical accomplice to DEN-prompted cancer-causing impacts (Shaarawy et al. 2009). LPO is a vital biomarker of oxidative damage in light of the fact that its levels are increased ROS production and assumes a fundamental part in the carcinogenesis actuated by DEN (Banakar et al. 2004; Vasquez-Garzon et al. 2009). MDA has for quite some time been utilized as a particular biomarker of oxidative harm and LPO levels; increments in MDA reflect upgrades of LPO (Lykkesfeldt 2007). In the present study, we found that the MDA levels were essentially raised by DEN. Also, the rats that were pretreated with MOLEE demonstrated a critical decrease in the level of MDA compared with the level observed in the DEN group. The observed decrease in the level of LPO in MOLEE-treated animals was apparently because of the increase in antioxidative abilities.

Thus, to counteract cell harm induced by ROS, an animal has numerous antioxidative barrier frameworks, including non-enzymatic (for the most part GSH) and enzymatic cancer prevention agent guards (counting SOD, GR, GST, CAT and GPx). GSH assumes an imperative part in keeping up the typical reducing state of cells and neutralizing the destructive impacts of oxidative damage (Ramakrishnan et al. 2006). GPx universally exists both in the cytosol and in the mitochondria of hepatocytes. GST is limited to the cytosol and assumes an essential part in the detoxification and discharge of xenobiotics using GSH (Bansal et al. 2005). Notwithstanding GSH and GSH-related antioxidant enzymes, other cell reinforcement compounds, including SOD and CAT, can likewise assume important roles in the cancer prevention agent protection framework. SOD can catalyze the dismutation of two superoxide radicals to H_2_O_2_ and O_2_. CAT goes about as a supporting antioxidant enzyme by changing H_2_O_2_ to H_2_O, along these lines giving assurance against ROS (Vasquez-Garzon et al. 2009). In the present study, DEN resulted in significant decrease in the level of GSH and the activities of antioxidation enzymes which may have resulted from the excessive LPO generated during the metabolism of DEN. However, the effects of DEN were partially counteracted by MOLEE, which suggests that the elevation in GSH and related antioxidant enzymes may be one of the important mechanisms of action of MOLEE against DEN-induced HCC (Saalu et al. 2011). The past studies exhibited that the total phenolic substance and the total flavonoid substance of *Moringa* leaves are ∼2- and 3-fold those of vegetables, separately (Pakade et al. 2013). Phenolic and flavonoid substance are known to be specifically connected to antioxidant activities (Siddhuraju & Becker 2003). The phenolic and flavonoid mixes of *Moringa oleifera* exert their antioxidant activities through scavenging free radicals, restraining enzymatic frameworks and metal chelation (Lukacinova et al. 2008; Sadek 2014).

Tumour initiation, metastasis, and maintenance generally are mediated by changes in apoptosis-related proteins. The past studies have exhibited that misregulation of apoptosis is an essential reason for HCC progression (Fabregat 2009). Thus, past reports demonstrated that the enlistment of apoptosis is an imperative event in the chemoprevention of tumours by normally occurring dietary substances (Khan et al. 2010). Thus, we assessed the alterations in the gene expression levels of apoptosis-related proteins. The Bcl-2 family, a known group of antiapoptotic proteins, act by neutralizing the proapoptotic proteins such as Bax protein and assume a critical part in guiding the tumour cells to undergo apoptosis (Tse et al. 2008). Bax is regularly present in the cytosol and moved to the mitochondria to initiate apoptosis, yet the action of Bax is overcome by antiapoptotic proteins, for example, Bcl-2 and Bcl-xL (Edlich et al. 2011). A considerable number of cells escape from apoptosis through up-regulation of Bcl-2 and Bcl-xL expression (Danial & Korsmeyer 2004). In the present study, MOLEE up-regulated the expression of Bax and down-regulated the expressions of Bcl-2 and Bcl-xL compared with the levels observed in the DEN group. The proportions of Bcl-2 to Bax as opposed to the levels of the individual proteins are thought to be basic in deciding cell survival or demise (Fukamachi et al. 1998). For instance, a diminished Bcl-2/Bax proportion will promote apoptosis (Gardner 2004). The present study showed that the proportion of Bcl-2 to Bax was unmistakably diminished by MOLEE, which may have prompted apoptotic reactions amid hepatocarcinogenesis induced by DEN. Previous studies have similarly showed that one of the essential components by which capsaicin, zerumbone, matrine and leptin anticipate HCC includes decreasing the Bcl-2/Bax proportion (Ma et al. 2008). The execution of apoptosis will be performed by caspase actuation. The initiation of caspase-3 is a typical event in two noteworthy pathways, i.e., the extrinsic and intrinsic pathways (Sarada et al. 2008). In the current study, DEN administration prevented the expression of caspase-3, while MOLEE activated the expression of caspase-3. The apoptosis induced by PTX-2 in human HCC cells is associated with the up-regulation of Bax and the activation of caspase-3 and the down-regulation of Bcl-2 and Bcl-xL (Qi et al. 2012). Along these lines, MOLEE initiated apoptosis by means of down-regulation of Bcl-2 and Bcl-xL and up-regulation of Bax and caspase-3 might be among the vital mechanisms of averting HCC. The β-arrestin-2 was first uncovered as an eliminator of G protein-coupled receptor signaling and has since been found to show novel capacities as a signal transducer in various signalling pathways (DeWire et al. 2007). Among these procedures, β-arrestin-2 has been found to block against apoptotic signalling through inactivation of the intrinsic apoptotic pathway (Khan et al. 2011). The present results revealed that β-arrestin-2 expression increases in DEN-treated rats may prompt an antiapoptotic effect, which concurs well with the upregulation of Bcl-2 and Bcl-xL and the downregulation of Bax and caspase-3. Then again, MOLEE treatment prompted a reduction in β-arrestin-2 mRNA expression levels and hence advanced apoptotic signalling, as proven by the upregulation of Bax and caspase-3. Moreover, β-arrestin-2 provides the Wnt signalling pathway (Bryja et al. 2007), which is oftentimes imbalanced in HCC.

It should be considered that one of the mechanisms of some exogenous chemicals that induce apoptosis is also increasing ROS production; in this way, chemicals that induce antioxidation produce toxic to apoptotic impacts, hypothetically. Also, a few chemicals hinder the growth by implementing antioxidant activity and at the same time initiating apoptosis. Case in point, polyphenols and saffron show noteworthy chemopreventive impacts against tumour by means of antioxidant activation and apoptosis acceptance (Amin et al. 2011). Consequently, it is not questionable that MOLEE indicated powerful antioxidant properties in addition to star apoptotic impacts. Pre-treatment with MOLEE decreased the generation of ROS; however, it did not prevent the apoptotic activities of MOLEE, which suggests that several mechanisms of HCC counteractive action are induced by MOLEE.

## Conclusions

It can be inferred that the results of the present study are the first to show the *in vivo* chemoprotective effect of MOLEE against DEN-induced HCC. The improvement of antioxidant activity and the enlistment of apoptosis were fundamental to the chemoprotective impact of MOLEE. Understanding that there are no successful treatment measures for HCC, our outcomes recommend the potential utilization of MOLEE in chemoprevention of HCC.
